# Supplementary material for: Deletion of ARPKD-associated Pkhd1 gene in mice results in decreased Tfap2b expression and eye abnormalities
Source: Nat Commun. 2026 Jul 23;17:8650. doi: 10.1038/s41467-026-75698-y (PMC13490598; doi:10.1038/s41467-026-75698-y)
Supplement: Supplementary file 2 [file 41467_2026_75698_MOESM2_ESM.pdf]

Description of Additional Supplementary Files

Supplementary Data 1: POAG_common-variant-all_gene-level

Supplementary Data 2: Hugeamp_poag_single-variant-associations

Supplementary Data 3: Primers used for mouse genotyping
