# Supplementary material for: Deletion of ARPKD-associated Pkhd1 gene in mice results in decreased Tfap2b expression and eye abnormalities
Source: Nat Commun. 2026 Jul 23;17:8650. doi: 10.1038/s41467-026-75698-y (PMC13490598; doi:10.1038/s41467-026-75698-y)
Supplement: Supplementary file 3 [file 41467_2026_75698_MOESM4_ESM.pdf]

nature portfolio

Corresponding author(s): Germino

Last updated by author(s): May 5, 2026

Reporting Summary

Nature Portfolio wishes to improve the reproducibility of the work that we publish. This form provides structure for consistency and transparency in reporting. For further information on Nature Portfolio policies, see our Editorial Policies and the Editorial Policy Checklist.

Statistics

For all statistical analyses, confirm that the following items are present in the figure legend, table legend, main text, or Methods section.

n/a	Confirmed	
☐	☒	The exact sample size (*n*) for each experimental group/condition, given as a discrete number and unit of measurement
☐	☒	A statement on whether measurements were taken from distinct samples or whether the same sample was measured repeatedly
☐	☒	The statistical test(s) used AND whether they are one- or two-sided<br>*Only common tests should be described solely by name; describe more complex techniques in the Methods section.*
☒	☐	A description of all covariates tested
☒	☐	A description of any assumptions or corrections, such as tests of normality and adjustment for multiple comparisons
☐	☒	A full description of the statistical parameters including central tendency (e.g. means) or other basic estimates (e.g. regression coefficient) AND variation (e.g. standard deviation) or associated estimates of uncertainty (e.g. confidence intervals)
☐	☒	For null hypothesis testing, the test statistic (e.g. *F*, *t*, *r*) with confidence intervals, effect sizes, degrees of freedom and *P* value noted<br>*Give P values as exact values whenever suitable.*
☒	☐	For Bayesian analysis, information on the choice of priors and Markov chain Monte Carlo settings
☒	☐	For hierarchical and complex designs, identification of the appropriate level for tests and full reporting of outcomes
☒	☐	Estimates of effect sizes (e.g. Cohen's *d*, Pearson's *r*), indicating how they were calculated

Our web collection on statistics for biologists contains articles on many of the points above.

Software and code

Policy information about availability of computer code

Data collection	For data collection, we used sratoolkit v.3.0.5; a Zeiss 800 confocal microscope; a Nikon CSU-W1 SoRa Spinning Disk Confocal Microscope; a Zeiss LSM700; a Keyence BZ-810; Zeiss 880 Confocal microscope;
Data analysis	For data analysis we used the following: bwa-mem2 v.2.2.1, cnvnator v.0.4.1, cellranger v.7.1.0, samtools v.1.17, R v.4.3.0, Seurat v.4.3., ggplot2 v.3.4.2, Integrative Genomics Viewer v.2.11.9, ImageJ v.1.54p, Prism 10.4.1, FIji Software, TFmotifView; code used to produce the data and images in Figures 1a, Figure 2i, 2j, Suppl Fig 4, Figure 5 and Suppl Figure 6 is available with all other data at Figshare with the following accession number 10.6084/m9.figshare.25970203.

For manuscripts utilizing custom algorithms or software that are central to the research but not yet described in published literature, software must be made available to editors and reviewers. We strongly encourage code deposition in a community repository (e.g. GitHub). See the Nature Portfolio guidelines for submitting code & software for further information.

Data

Policy information about availability of data

All manuscripts must include a data availability statement. This statement should provide the following information, where applicable:

- Accession codes, unique identifiers, or web links for publicly available datasets
- A description of any restrictions on data availability
- For clinical datasets or third party data, please ensure that the statement adheres to our policy

All data and relevant metadata described in the results or presented in the figures and supplementary figures of this study have been deposited in Figshare with the

following accession numbers 10.6084/m9.figshare.25970203 and will be available immediately.

Research involving human participants, their data, or biological material

Policy information about studies with human participants or human data. See also policy information about sex, gender (identity/presentation), and sexual orientation and race, ethnicity and racism.

Reporting on sex and gender	N.A.
Reporting on race, ethnicity, or other socially relevant groupings	N.A.
Population characteristics	N.A.
Recruitment	N.A.
Ethics oversight	N.A.

Note that full information on the approval of the study protocol must also be provided in the manuscript.

Field-specific reporting

Please select the one below that is the best fit for your research. If you are not sure, read the appropriate sections before making your selection.

[x] Life sciences [ ] Behavioural & social sciences [ ] Ecological, evolutionary & environmental sciences

For a reference copy of the document with all sections, see nature.com/documents/nr-reporting-summary-flat.pdf

Life sciences study design

All studies must disclose on these points even when the disclosure is negative.

Sample size	Sample sizes were not pre-calculated.
Data exclusions	Histologic samples that were either incorrectly embedded or sectioned were not analyzed.
Replication	Most studies were done with multiple replicates, as reported in the manuscript with the n described. The only exception to this is the RNAscope studies done of the liver as a positive control where a single liver was analyzed with a positive and negative control probe.
Randomization	not relevant, as we are comparing the available samples of the relevant genotypes
Blinding	in the histological analyses, the investigator doing the measurements was blinded to genotypes.

Reporting for specific materials, systems and methods

We require information from authors about some types of materials, experimental systems and methods used in many studies. Here, indicate whether each material, system or method listed is relevant to your study. If you are not sure if a list item applies to your research, read the appropriate section before selecting a response.

Materials & experimental systems

n/a	Involved in the study	
[ ]	[x]	Antibodies
[x]	[ ]	Eukaryotic cell lines
[x]	[ ]	Palaeontology and archaeology
[ ]	[x]	Animals and other organisms
[x]	[ ]	Clinical data
[x]	[ ]	Dual use research of concern
[x]	[ ]	Plants

Methods

n/a	Involved in the study	
[x]	[ ]	ChIP-seq
[x]	[ ]	Flow cytometry
[x]	[ ]	MRI-based neuroimaging

Antibodies

Antibodies used	: anti-Sox10 (1:100, 2ug/ml) sc365692 Santa Cruz Biotechnology; anti-AP-2β (1:100) cst2509 Cell Signaling Technology; anti-Sox9 ab5535 (1:500, 0.002ug/ml); anti-ZO-1 (1:100, 2.5ug/ml) #40-2200 Invitrogen); anti-smooth muscle actin (AB5694, 1:300, Abcam); anti-RBPMS (ABN1376, ABN1362 1:300, Millipore Sigma); anti-Pax6 (PRB-278P, 1:300, Covance). The following secondary antibodies used for frozen sections were obtained from Thermo Fisher Scientific: donkey anti-rabbit Alexa fluor 488 (1:1000), Alexa555-anti-mouse IgG, donkey anti-mouse Alexa fluor 568 (1:1000), donkey anti-rabbit Alexa fluor 647 (1:1000). Either DAPI or Hoechst 33342 were used for nuclear staining.
Validation	anti-Sox10 (sc365692, Santa Cruz Biotechnology) Immunoblot of multiple cell lines (SK-MEL-24, C6, A-375); multiple publications citing use.<br>anti-AP-2β (cst2509, Cell Signaling Technology) Immunoblot of multiple cell lines (SH-SY5Y, U-87MG, U-118MG) and immunofluorescent analysis of SH-SY5Y cells; multiple publications citing use.<br>anti-Sox9 (ab5535, Millipore Sigma) Immunoblot of HepG2 cell lysate; multiple publications citing use.<br>anti-ZO-1 (#40-2200, Invitrogen, RRID AB_2533456) Immunoblot and immunofluorescent testing in multiple cell types and tissues; advanced verification using cell perturbations and siRNA; multiple publications citing use.<br>anti-smooth muscle actin (AB5694, Abcam) No validation described by the supplier but multiple publications citing use with many images demonstrating specificity for immunofluorescence and immunoblot use.<br>anti-RBPMS (ABN1376, Millipore Sigma) Immunoblot of mouse embryonic stem cell (mESC) lysate; multiple publications citing use.<br>anti-RBPMS (ABN1362, Millipore Sigma) Immunoblot of mouse embryonic stem cell (mESC) lysate. multiple publications citing use.<br>anti-Pax6 (PRB-278P, Covance, RRID AB_291612) Immunoblot of multiple cell and tissue extracts and immunohistochemical staining of tissue; multiple publications citing use.

Animals and other research organisms

Policy information about studies involving animals; ARRIVE guidelines recommended for reporting animal research, and Sex and Gender in Research

Laboratory animals	Mouse, C57BL/6N, ages embryonic date 8 to over 1 year. Lines of mice: Pkhd1del3-67; Tfap2bko
Wild animals	No wild animals were used in this study.
Reporting on sex	Our study examined male and female animals and found no differences at the time points studied. The results are described in the text and presented in the supplemental figures. We have also included the sex of the animal for specimens in the source data files that will be publicly shared with the report.
Field-collected samples	no field-collected samples were used in this study.
Ethics oversight	Mouse studies were performed under protocols K001-KDB-19, K001-KDB-22 and NEI 556 which had been approved by the NIH Animal Care and Use Committee. Mice were housed in pathogen-free animal facilities accredited by the American Association for the Accreditation of Laboratory Animal Care and meet Federal (NIH) guidelines for the humane and appropriate care of laboratory animals.

Note that full information on the approval of the study protocol must also be provided in the manuscript.

Plants

Seed stocks	Not applicable
Novel plant genotypes	Not applicable
Authentication	Not applicable
