# Supplementary material for: Deletion of ARPKD-associated Pkhd1 gene in mice results in decreased Tfap2b expression and eye abnormalities
Source: Nat Commun. 2026 Jul 23;17:8650. doi: 10.1038/s41467-026-75698-y (PMC13490598; doi:10.1038/s41467-026-75698-y)
Supplement: Supplementary file 4 [file 41467_2026_75698_MOESM5_ESM.pdf]

nature portfolio

Peer Review File

Deletion of ARPKD-associated *Pkhd1* gene in mice results in decreased *Tfap2b* expression and eye abnormalities

Corresponding Author: Dr Gregory Germino

This file contains all reviewer reports in order by version, followed by all author rebuttals in order by version.

A version of this paper was originally rejected for publication by Nature Communications, however that decision was reconsidered after appeal by the authors.

Version 0:

Reviewer comments:

Reviewer #1

(Remarks to the Author)
Ishimoto et al present a fascinating observation and dissection of an unexpected glaucoma phenotype in mice homozygous for an engineered Pkhd1 mutation (Pkhd1<del3-67>). This could not be complemented with a Tfap2b null mutation, supporting the hypothesis that this phenotype was due to a lack of Tfap2a activity. The authors propose that this may be due to the loss of a Tfap2b regulatory element within Pkhd1, although this was not conclusively demonstrated.

The phenotypic characterisation is thorough, and it is clear from this paper, and others, that Tfap2b is the most likely causal locus for the glaucoma phenotype. The paper is well-written.

My key question surrounds the nature of the engineered allele and background strain, and ensuring that the Pkhd1 exon 3-67 deletion truly is the only relevant variant here. It would be important to look across this genomic interval to confirm that this was the case, and to exclude the possibility that another variant was not inherited or introduced at the Tfap2b locus during or after Pkhd1 mutagenesis. This could be done e.g. by performing WGS and variant calling on both mutant and wild-type mice. I note that WGS was performed in the original paper to confirm the Pkhd1 deletion in homozygous mutants.

The authors demonstrate that Tfap2b RNA is highly expressed in mouse corneal stroma (Fig 3f), although do not compare expression between wild-type and del3-67 heterozygotes and homozygotes (or compound heterozygotes). This would be an important piece of evidence to support the hypothesis of disruption of a transcriptional regulation as the causal mechanism.

Otherwise it is somewhat unclear why AP-2B is detected by immunostaining at E8 and E9, but not at E13.5 or beyond. Can the authors please delve into this a bit more, including consideration of the reagents used to detect it. Could this be explained by different RNA or protein isoforms, and if so, could the authors test this hypothesis with different antibodies or RNA sequencing?

Minor points

in the methods, please clarify the breeding strategy and if mice used in experiments were littermates

Figure 2: rebound tonometry is heavily influenced by corneal thickness. this should be presented alongside the IOP data, for example by measuring the central corneal thickness by pachymetry and/or histopathology

Fig 2 & 6 etc - in several figures data from both eyes were included in the analysis, although it was not clear how this non-independence was dealt with statistically. If this could be articulated in the figure and/or methods please.

line 133 - it would be preferable to be more specific when referring to Pkhd1 mutants (e.g. be explicit if referring to del3-67 homozygotes)

lines 170 and 172 - some additional detail around the difference between Sox9 and Sox10 would be useful here

Reviewer #2

(Remarks to the Author)
In this manuscript by Ishimoto et al. the authors provide a compelling story suggesting that a large deletion of most of the Pkhd1 gene in mice results in disruption of cis-regulation in a spatiotemoporal fashion of the nearby gene Tfap2b which is necessary for eye development, thereby resulting in a model of early onset glaucoma with 100% penetrance. The authors have appropriate multidisciplinary expertise including Pkhd1 gene and genomics as well as eye pathology. The Tfap2b gene has been implicated in this eye phenotype in a mouse model with a complex genomic rearrangement in the region of Tfap2b. Indeed this manuscript demonstrates that Tfap2b recessive mice die perinatally, but those that survive have a very similar eye phenotype. Notably another investigation of Tfap2b recessive genotype on a different genetic background allowed mice to survive longer post-natally and while they died of renal failure at young age the authors of that study did not mention noting eye abnormalities. The novelty of this manuscript is the implication of potential cis-regulation of Tfap2b by the genomic region containing Pkhd1 exons 3-67, and how this could contribute an explanation of the GWAS peaks in this region that have previously been unexplained. Evidence for the proposed cis-regulatory mechanism in a topographically associated domain is shown with data showing absent Tfap2b (AP-2B)protein in relevant compartments of the eye in the Pkhd1 del3-67 recessive mice, while (per supplemental data) still present in other regions. And, a similar phenotype between in Tfap2b het;Pkhd1 del3-67 het mice as recessive for either gene.

Overall, the investigation is elegantly done with appropriate controls, dataset sizes/genders, analysis. The authors appropriately avoid overstating conclusions. Shortcomings to its impact are the lack of validation in an independent model and only possible but not definitive human relevance. Indeed, the genomic locus including the Pkhd1 and Tfab2b genes maintains the same relative orientation and similar distance between these genes in mouse compared to human, and human SNPs in this region have been implicated in multiple GWAS studies for adult onset open angle glaucoma (different than the mouse phenotype but perhaps acceptably so based on other models showing similar species differences). The fact that many of the human SNPs implicated in this region are protective as opposed to deleterious seems less supportive of the proposed mechanism.

Comments:
--The title appropriately (based on available data) conveys some degree of remaining uncertainty of mechanism in this particular mouse model and its reproducibility and human relevance. Without an independent model validation it will be difficult to 100% confirm the mechanism and refute the chance of other cis-regulatory effects specific to their genomic manipution that generated the Pkhd1 del 3-67 allele. This uncertainty unfortuantely lowers the significance of the manuscript and makes the findings somewhat descriptive of this particularly model.
--the genomic position of the boundaries of the Pkhd1 deletion should be provided even if previously published
--While it seems less likely, one possibility that the authors don't appear to investigate fully but could do better to exclude is that their excision of Pkhd1 exons 3-67 resulted in a larger than expected genomic disruption/rearrangement that disrupted the Tfap2b gene or its more proximal regulatory factors. Description of what sequence/probe validation confirmed the deletion of Pkhd1 exons and the presence of wild-type tfab2b on this allele should be provided. Long-read sequencing may provide more reassurance that the allele is fully intact other then the intended deletion. A more deliberate demonstration of successful Tfap2b expression in other spatiotemporal conditions would alternatively be reassuring that the Tfap2b gene is not disturbed.
--single cell or tissue specific RNA seq could be informative to validate the proposed transcriptional regulation and distinguish that the lack of AP-2B staining in certain structures by immunoflourescence is indeed due to cis-regulation from the Pkhd1 genomic region as opposed to effects from different cell type/differentiation/microenvironment in the tested region as it develops abnormaly.

minor comments:
--Figure 3 provides publicaly available data only relevant to an alternative hypothesis and perhaps could thus be supplemental.
--Figure 1c would benefit from added information for the mouse allele such as chromosome number, (+/-) strand, relative genomic distances.
--Expression of Pkhd1 in eye is not commonly known - often thought to be kidney, liver, pancreas specific. If more than one dataset shows transcriptomic data from eye relative to kidney that should be provided. Alternatively, if the Pkhd1 expression is not thought relevant in the proposed model which it seems no to be, that should be made more clear.
--Supplemental Figure 6: Given the equivalency between the on-target and negative control, it is not clear that the RNA scope produces any meaningful positive or negative data on eye tissue. However, as this was to investigate an alternative hypothesis, this may be ok, although doesn't contribute to excluding the alternative hypothesis of a Pkhd1 shortened transcript playing a regulatory role.

Version 1:

Reviewer comments:

Reviewer #2

(Remarks to the Author)
Since the original submission, the authors have provided additional data/analyses/discussion which is largely supportive of their initial claims and satisfy my concerns. Their rebutal is well conceived. The implication that the region deleted in the author's Pkhd1 del 3-67 model affects expression of Tfap2b in cis, as opposed to other off-target effects from making the allele, is now well supported. The case for a causative effect between loss of Tfap2b expression and the glaucoma phenotype reported is also strong. Together, this allows for an interesting manuscript with sufficient evidence to implicate the genomic deletion of the Pkhd1 exon 3-67 in regulation of the Tfap2b locus in a way relevant for normal eye development. Their case for this being the explanation for association of human SNPs in this region with glaucoma phenotype is strong albeit not granularly defined. The authors propose a logical and supported model in Figure 7 and include some hypotheses as it remains undefined exactly where and how in this region the relevant interaction takes place.

The revision added analysis of WGS for the Pkhd1 del 3-67 allele to address the concern from both reviewers regarding whether anything else in LD with the Pkhd1 del 3-67 locus could provide an alternative explanation. The authors consider several possibilities that they entertained and give a thorough discussion in the rebutal. They reviewed WGS on homozygous Pkhd1 del 3-67 mice for point mutations, CNVs, and visually inspected reads in IGV for structural variants but found no additional variants in the region of the Tfap2b locus. The WGS data with zoomed out view from IGV now provided in Supp Figure 9, excludes any large deletion at the Tfap2b locus and I trust that inspection was done thoroughly in the Tfap2b region to consider any SNVs affecting the Tfap2b promotor or breakpoints for translocations or other disrupting variants that could be otherwise missed by WGS variant callers. It comes across looking a bit surprising that color bars in the figure could suggest heterozygous variants in this homozyogus region but I trust this is just the nature of uncurated WGS data. Figure 1 has been much improved now including genomic positions. I am satisfied with this work.

To address another concern shared by both reviewers, the authors used RNA scope to illustrate the marked reduction in Tfap2b mRNA expression in the relevant cells of the homozygous Pkhd1 del 3-67 mice. This adds a complementary assessment of what was found by immunostaining, and is nicely convincing that Tfap2b expression is dramatically reduced at the relevant timepoint and cell type in these mice, and Figure 3 is improved to show this.

In summary, the investigation supports the claims and will be a valuable contribution to the literature.

minor comments:
--The first paragraph of the discussion now nicely highlights additional supporting data that an independent disruption of this locus by other investigators (who were targeting XDH) resulted in the same phenotype. This is good to highlight, but the wording may be confusing at first glance. As written, it sounded like the authors of this manuscript had intended to target XDH.

--the edited paragraph header "Pkhd1 is not expressed in the developing eye" feels almost contradictory to the sentences that follow and provided data (supp fig 4a) which appears to show some localized expression, albeit perhaps not in the appropriate context. Perhaps the authors intended to state such a definitive conclusion in light of the clear result presented in "Figure 1 for reviewers", however that isn't part of the manuscript as is. The Figure 1 for reviewers and other scRNA seq data would benefit from clarifying the age at which they were tested if the authors consider that relevant. Nonetheless, while the investigation for a transcript consisting of exons 54-56 is not exhaustive, this seemed an unlikely possibility so it is adequate, and the authors supposition that Pkhd1 expression is not relevant seems a reasonable claim from the data.

--Figure 7 has been improved by the added illustration (7a). However, it looks like the Tfap gene illustration was inadvertently left off (7c wild-type allele 2). This is just a typo but doesn't change interpretation.

Reviewer #4

(Remarks to the Author)
The strongest SNP associated with POAG in GWAS studies, rs17752199, is located between Tfap2b and Pkhd1. It is well known that Tfap2b is involved in anterior segment development. The conditional deletion of Tfap2b in mouse cranial neural crest results in anterior segment dysgenesis and congenital glaucoma. In the current study the authors show that a large deletion that includes Pkhd1 results in anterior segment dysgenesis and congenital glaucoma with concurrent suppression of Tfap2b. They hypothesize that “that gene expression of Tfap2b is regulated by at least two spatiotemporal tissue-specific enhancers and that cis-acting genomic interactions with one of them is disrupted by the Pkhd1 del3-67 deletion.” This is consistent the genetic alterations with other ocular abnormalities. PAX6 mutations cause aniridia. The SIMO element is a distal transcriptional enhancer located within the first intron of the downstream ELP4 gene which enhances the expression of the Pax6 gene. Genetic alterations in SIMO result in loss of Pax6 expression and aniridia. In the same fashion, the Pkhd1 del3-67 deletion could be eliminating a distal regulatory region for Tfap2b.

Unfortunately, there is no direct data identifying any regulatory elements contained inside the Pkhd1 del3-67 deletion and there is no reason to believe that there is any direct connection between Pkhd1 genetic alterations and the SNPs located in this region that modify risk to POAG. The effect of deleting Pkhd1 in these mice results in decreased expression of Tfap2b and this change in expression results in the observed known phenotype in the mice. However, the genetic abnormality altering Tfap2b expression in patients with POAG could easily be elsewhere, such as the region between Tfap2b and Pkhd1 or in the Tfap2b gene itself.

The main finding in this paper is that there may be a regulatory region for Tfap2b inside Pkhd1 but it has not been further

delineated by these experiments. Where is it? What is the mechanism? Otherwise, there is little novelty in confirming that an anterior segment dysgenesis phenotype can be caused by the loss of Tfap2b.

With regards to the previous review of this manuscript, reviewer 1 stated “It would be important to look across this genomic interval to confirm that this was the case, and to exclude the possibility that another variant was not inherited or introduced at the Tfap2b locus during or after Pkhd1 mutagenesis. This could be done e.g. by performing WGS and variant calling on both mutant and wild-type mice.” This is a valid concern. While the authors provide some rebuttal for why they believe another variant isn’t responsible, the issue cannot be eliminated without the requested WGS and variant calling on both mutant and wild-type mice, which has not been done.

Version 2:

Reviewer comments:

Reviewer #4

(Remarks to the Author)
The authors say in their rebuttal “In fact, GWAS studies unequivocally associate Primary Open Angle Glaucoma (POAG) to PKHD1 and not TFAP2B.”

This is not correct and the authors are not citing the most recent GWAS studies. Han et al 2025 (Nature Genetics) find SNPs are associated with both PKHD1 and TFAP2B. There is one significant SNP that is intronic in each gene and one that is intergenic. Their co-localization studies did not support either gene. Gene based analysis support both genes with a stronger result for TFAP2B. Fine mapping did not support either gene. Thus the GWAS data does not support one gene over the other. Additionally, only half of the time is the gene nearest to a SNP the one responsible for the effect.

What is known is that deletions of either gene in mice are associated with anterior segment developmental defects. Recessive mutations in humans in each gene have not been associated with POAG or any other form of glaucoma. They state that “What we claim is: In this study, we provide in vivo evidence showing that genomic elements within or near Pkhd1 control Tfap2b expression in a very limited set of neural crest-derived cells in the POM, and the latter is responsible for the corneal endothelial and stromal abnormalities that were observed.” They should focus on presenting their results and not on explaining the human POAG GWAS association data. The proper title for the paper should be “Deletion of Pkhd1 in mice is associated with decreased Tfap2b expression and corneal abnormalities.”

Lastly Figure 1a is wrong. Their Manhattan plot does not reflect the association values of these loci for POAG on the website cited #37. For instance, the strongest SNPs for POAG GWAS is CDKN2B not LOXL1. Additionally, the authors should cite the primary literature here not a compilation website.

Version 3:

Reviewer comments:

Reviewer #4

(Remarks to the Author)
Article is acceptable in last revision.

Open Access This Peer Review File is licensed under a Creative Commons Attribution 4.0 International License, which permits use, sharing, adaptation, distribution and reproduction in any medium or format, as long as you give appropriate credit to the original author(s) and the source, provide a link to the Creative Commons license, and indicate if changes were made.
In cases where reviewers are anonymous, credit should be given to 'Anonymous Referee' and the source.
The images or other third party material in this Peer Review File are included in the article's Creative Commons license, unless indicated otherwise in a credit line to the material. If material is not included in the article's Creative Commons license and your intended use is not permitted by statutory regulation or exceeds the permitted use, you will need to obtain permission directly from the copyright holder.
To view a copy of this license, visit https://creativecommons.org/licenses/by/4.0/

REVIEWER COMMENTS

Reviewer #1 (Remarks to the Author):

Ishimoto et al present a fascinating observation and dissection of an unexpected glaucoma phenotype in mice homozygous for an engineered Pkhd1 mutation (Pkhd1). This could not be complemented with a Tfap2b null mutation, supporting the hypothesis that this phenotype was due to a lack of Tfap2a activity. The authors propose that this may be due to the loss of a Tfap2b regulatory element within Pkhd1, although this was not conclusively demonstrated.

The phenotypic characterisation is thorough, and it is clear from this paper, and others, that Tfap2b is the most likely causal locus for the glaucoma phenotype. The paper is well-written.

My key question surrounds the nature of the engineered allele and background strain, and ensuring that the Pkhd1 exon 3-67 deletion truly is the only relevant variant here. It would be important to look across this genomic interval to confirm that this was the case, and to exclude the possibility that another variant was not inherited or introduced at the Tfap2b locus during or after Pkhd1 mutagenesis. This could be done e.g. by performing WGS and variant calling on both mutant and wild-type mice. I note that WGS was performed in the original paper to confirm the Pkhd1 deletion in homozygous mutants.
Response 1.1: We are very appreciative of the reviewer's positive comments. We also agree that it is important to demonstrate that the complicated Cre-mediated deletion event that created the exon 3-67 deletion did not disrupt the *Tfap-2b* locus or adjacent sequences. If it had, the modified genomic region would segregate with the *Pkhd1*$^{del3-67}$ haplotype and would make it very difficult to determine whether the observed effects were from the *Pkhd1*del3-67 deletion or the modified *Tfap2*b allele since both would complement the *Tfap2b* null allele. This issue was also raised by Reviewer #2, highlighting the importance of this foundational finding.

We offer several lines of evidence to support our conclusion that any observed effects are solely due to the *Pkhd1* deletion and no other "off-target" effects or strain variants:

1. Neither original strain of mice used to produce the *Pkhd1*$^{del3-67}$ mouse line (ie *Pkhd1*$^{del3-4/del3-4}$, *Pkhd1*$^{del6/del677}$) had an eye phenotype. Therefore, any sequence variants, microdeletions, or other strain differences inherent to the lines cannot explain our observations.
2. Since we did not breed the *Ddx4*-Cre line to homozygosity, we can't absolutely exclude that it could have had a variant in its *Tfap2b-Pkhd1* haplotype that affects *Tfap2b* expression when present in the homozygous state. This is extremely unlikely to be relevant for our findings, however:
    a. We obtained the line directly from Jackson Laboratory, they report that hemizygous mice may be bred together or to wildtype siblings, and they do not

report an eye phenotype. This phenotype is not subtle and highly unlikely to have been missed by animal care personnel.

b. The proximity of the *Tfap2b* and *Pkhd1* loci makes it very unlikely that recombination occurred between the *Tfap2b* locus on the *Ddx4-Cre* haplotype and either *Pkhd1*$^{del3-4}$ *or Pkhd1*^{del67} locus, creating a new haplotype that subsequently maintained a variant from the *Ddx4-Cre* line in the *Pkhd1*$^{del3-67}$ mouse lines.
c. As described in our original report of the *Pkhd1*$^{del3-67}$ line [1], we generated 4 independent *Pkhd1*$^{del3-67}$ founders and bred them separately to homozygosis. All independently generated lines had overlapping phenotypes, including the eye anomalies. This finding indicates that the phenotype is unlikely the result of a random, stochastic effect of the *Ddx4-cre* on chromosome structure.
d. To further confirm that the line did not carry additional changes other than the intended deletion, in our initial characterization of the *Pkhd1 del3-67 deletion* we performed whole genome sequencing (WGS) of a homozygous *Pkhd1*$^{del3-67/del3-67}$ mouse DNA sample using Illumina Platform, 150bp paired-end. Reads were processed using BWA-MEM2 [2] and samtools [3]. Visual inspection using Integrative Genomics Viewer [4] confirmed absence of reads in the intended genomic region (chr1:20,053,820-20,613,713) and presence of reads in nearby genomic intervals, including the *Tfap2b* genomic region. Structural variation analysis using CNVnator [5] identifies a single, large genomic deletion in the *Pkhd1* locus but no changes in the *Tfap2b* locus. We have added a figure showing this to the supplement (Suppl Figure 9), and we have also added text explicitly stating this.

The authors demonstrate that Tfap2b RNA is highly expressed in mouse corneal stroma (Fig 3f), although do not compare expression between wild-type and del3-67 heterozygotes and homozygotes (or compound heterozygotes). This would be an important piece of evidence to support the hypothesis of disruption of a transcriptional regulation as the causal mechanism.
Response 1.2: This is another point raised by both reviewers. We have therefore performed RNAscope studies of the eyes of E13.5 Control, *Pkhd1*$^{del3-67/+}$, *Pkhd1*$^{del3-67/del3-67}$, *Pkhd1*$^{del3-67/+}$*; Tfap2b*^{ko} embyros and the results show that *Tfap2b* expression is markedly lower in both the *Pkhd1*$^{del3-67/del3-67}$ and *Pkhd1*$^{del3-67/+}$*;Tfap2b*$^{KO/}$mutant eyes than in the Control and *Pkhd1*$^{del3-67}$ heterozygotes. These data are now included in Figures 3e and 6k.

Otherwise it is somewhat unclear why AP-2B is detected by immunostaining at E8 and E9, but not at E13.5 or beyond. Can the authors please delve into this a bit more, including consideration of the reagents used to detect it. Could this be explained by different RNA or protein isoforms, and if so, could the authors test this hypothesis with different antibodies or RNA sequencing?
Response 1.3: We would like to respectfully correct one misunderstanding. The reviewer notes that AP-2β was detected only in early embryos (E8, E9) and not at later stages (E13.5). In fact, we do detect *Tfap2b* transcript and AP-2β in *Pkhd1*$^{del3-67/del3-67}$ and *Pkhd1*$^{del3-}$

$^{67/+}$; *Tfap2b*$^{ko/+}$mutants at E13.5 (Figure 3e, Supplementary Figure 7d and Supplementary Figure 12e), but expression is restricted to the peripheral periocular mesenchyme (POM) of the developing eye in both examples. These examples nicely show that the RNAscope probe and AP-2β antibody did work on the samples, but the pattern differed between WT and mutants.

So why the differential staining patterns? It cannot be due to the choice of antibody since we used the same antibody for <u>all</u> studies in this report. Our explanation for why the staining patterns differ in early vs later ages in mutant and control mice is that the *Pkhd1*del3-67 deletion disrupts *Tfap2b* expression in a spatiotemporally-restricted manner. There are several lines of evidence to support this:

1. Our *Pkhd1*$^{del3\text{-}67/del3\text{-}67}$ mice do not have the phenotypes previously reported with germline *Tfap2b* deletion. The latter generally die in the perinatal period, with survivors having polycystic kidney disease. In contrast, our *Pkhd1*$^{del3\text{-}67/del3\text{-}67}$ mice do not die in the perinatal period and, ironically, do not develop renal cystic disease. These observations are consistent with our data showing no difference in AP2β patterns in early embryos (Figure 4, Supplementary Figure 8).
2. *Tfap2b* is known to undergo a complex pattern of cell-type specific expression [6-8]. As noted in our report, conditional deletion of *Tfap2b* in neural crest cells using the Wnt1-cre results in a phenotype like ours.
3. And as described in **Response 1.1**, there is no evidence for a deletion affecting *Tfap2b* introns that could explain a splicing effect.

Minor points

in the methods, please clarify the breeding strategy and if mice used in experiments were littermates

Response 1.4: Littermates were used for each experiment and were generally the offspring of *Pkhd1* $^{del3\text{-}67/+}$ x *Pkhd1* $^{del3\text{-}67/+}$ or *Pkhd1* $^{del3\text{-}67/+}$ x *Tfap2b*$^{ko/+}$ mating, though we also used *Pkhd1* $^{del3\text{-}67del3\text{-}67+}$ (female) x *Tfap2b*$^{ko/+}$ (male) mating to increase the probability of getting trans-heterozygotes. We have added these details to the methods section of the revised manuscript.

Figure 2: rebound tonometry is heavily influenced by corneal thickness. this should be presented alongside the IOP data, for example by measuring the central corneal thickness by pachymetry and/or histopathology

Response 1.5: While corneal thickness correlates with IOP when measured by rebound tonometry in humans, it is less clear whether this is true in mice. The literature suggests that it does not correlate well in normal mice [9] or mice with transgenic mutations of matricellular proteins [10], though it is unknown if their conclusions would apply when the differences in corneal thickness are large [10].

In response to your request, we measured corneal thickness in eyes of 3-week-old control and *Pkhd1*$^{del3\text{-}67/del3\text{-}67}$ mice and confirmed that it was approximately 50% thicker in the mutants than in controls. We have added these data to Figure 2b. We acknowledge the uncertain effects that observed changes in corneal thickness and structure could have on measurements of IOP using rebound tonometry, and we have added text to the report acknowledging the complexity of interpreting high IOP under these circumstances. We note, however, that even if interpreting IOP measurements in our model is complicated by differences in thickness and/or composition, there was an acquired large reduction in Retinal Ganglion Cell (RGC) number that correlated highly with high IOP, and this is a known consequence of high IOP in glaucoma.

We also want to reassure the reviewer that we had formally excluded the *rd8* mutation (ie. a single nucleotide deletion in *Crb1*) as a contributing factor to the retinal disease that we observed. While the histopathology of eyes of the *Pkhd1*$^{del3\text{-}67/del3\text{-}67}$ mice is very different than the pattern of retinal degeneration associated with the *rd8* mutation, the latter is commonly found in the C57BL/6N background mouse strain of the *Pkhd1*$^{del3\text{-}67}$ mouse lines [11].

Fig 2 & 6 etc - in several figures data from both eyes were included in the analysis, although it was not clear how this non-independence was dealt with statistically. If this could be articulated in the figure and/or methods please.
Response 1.6: In most studies (IOP, corneal membrane and retinal thickness, eye weight/body weight), we used the mean values for both eyes, so each animal contributed only one value to the figure and analysis, which was done using Wilcoxon rank test (Mann-Whitney). For the RGC measurements, we compared the mean value from a single eye for each mouse. For eye weights, we compared the mean values for all eyes of each genotype, but we used the mean values for both eyes in the eye weight/body weight analysis. These details are now explicitly noted in both the figure legends and methods.

line 133 - it would be preferable to be more specific when referring to Pkhd1 mutants (e.g. be explicit if referring to del3-67 homozygotes)
Response 1.7: We have edited the text in multiple places accordingly.

lines 170 and 172 - some additional detail around the difference between Sox9 and Sox10 would be useful here
Response 1.8: Since we detected a loss of AP-2β expression in neural crest derivatives in the eye, and since AP-2β is broadly expressed in all NCC at the pre-migratory and migratory stages before they populate various target tissues, we asked whether NCC development was affected already during earlier embryonic stages in *Pkhd1*$^{del3\text{-}67/del3\text{-}67}$ mice. As neural crest cells are specified in the dorsal aspects of the neural tube, a cascade of transcription factors, including Sox9 and Sox10, are activated at different stages of the maturation process. Sox9 is expressed in the early pre-migratory NCCs whereas Sox10 is first activated when NCCs go through epithelial-mesenchymal transition to delaminate

from the neural epithelium. Sox10 can therefore be used as a marker for newly as well as fully migrating neural crest cells that enter different target organs. To fully cover both stages of neural crest development, we used these two markers accordingly to understand whether the neural crest at these pre-migratory and migratory stages was impacted in the *Pkhd1*$^{del3\text{-}67/del3\text{-}67}$ mutants. We examined the patterns of expression of AP-2β and Sox9 at E8 and found normal pre-migratory NCCs in *Pkhd1*$^{del3\text{-}67/del3\text{-}67}$ mutants comparable to WT littermates (Figure 4a). Similarly, a day later at E9, when Sox10-positive NCCs have reached their target organs, including the eye cup, we found that AP-2β -positive NCCs had normally populated the eye in both WT and *Pkhd1*$^{del3\text{-}67/del3\text{-}67}$ mutants (Figure 4b-h'). Additionally, the results showed AP-2β- positive NCCs were normally populating all other destination organs in *Pkhd1*$^{del3\text{-}67/del3\text{-}67}$ mutants (Supplementary Figure 8). Collectively, these results suggest that *Pkhd1* $^{del3\text{-}67/del3\text{-}67}$ mutants have normal neural crest development and *AP-2β* expression throughout the migrating stage, and that the NCC normally reach their target organs. However, once in the eye cup, the NCC-derived POM region no longer expresses *AP-2β* normally.

Reviewer #2 (Remarks to the Author):

In this manuscript by Ishimoto et al. the authors provide a compelling story suggesting that a large deletion of most of the Pkhd1 gene in mice results in disruption of cis-regulation in a spatiotemoporal fashion of the nearby gene Tfap2b which is necessary for eye development, thereby resulting in a model of early onset glaucoma with 100% penetrance. The authors have appropriate multidisciplinary expertise including Pkhd1 gene and genomics as well as eye pathology. The Tfap2b gene has been implicated in this eye phenotype in a mouse model with a complex genomic rearrangement in the region of Tfap2b. Indeed this manuscript demonstrates that Tfap2b recessive mice die perinatally, but those that survive have a very similar eye phenotype. Notably another investigation of Tfap2b recessive genotype on a different genetic background allowed mice to survive longer post-natally and while they died of renal failure at young age the authors of that study did not mention noting eye abnormalities. The novelty of this manuscript is the implication of potential cis-regulation of Tfap2b by the genomic region containing Pkhd1 exons 3-67, and how this could contribute an explanation of the GWAS peaks in this region that have previously been unexplained. Evidence for the proposed cis-regulatory mechanism in a topographically associated domain is shown with data showing absent Tfap2b (AP-2B) protein in relevant compartments of the eye in the Pkhd1 del3-67 recessive mice, while (per supplemental data) still present in other regions. And, a similar phenotype between in Tfap2b het;Pkhd1 del3-67 het mice as recessive for either gene.

Overall, the investigation is elegantly done with appropriate controls, dataset sizes/genders, analysis. The authors appropriately avoid overstating conclusions. Shortcomings to its impact are the lack of validation in an independent model and only possible but not definitive human relevance. Indeed, the genomic locus including the Pkhd1 and Tfab2b genes maintains the same relative orientation and similar distance between these genes in mouse compared to human, and human SNPs in this region have

been implicated in multiple GWAS studies for adult-onset open angle glaucoma (different than the mouse phenotype but perhaps acceptably so based on other models showing similar species differences). The fact that many of the human SNPs implicated in this region are protective as opposed to deleterious seems less supportive of the proposed mechanism.

Response 2.1: We thank the reviewer for their positive comments and welcome the opportunity to respond to their thoughtful queries. We would like to begin by noting that the *PKHD1* SNP most highly associated with POAG/IOP is associated with increased risk, as are two others (3/8 SNPs linked to *PKHD1* are associated with increased risk). Importantly, one of two *TFAP2B* SNPs also associated with POAG/IOP is associated with **decreased** risk. These observations strongly suggest that variants or structural changes in this interval can increase or decrease risk, presumably by affecting *TFAP2B* expression.

Comments:

--The title appropriately (based on available data) conveys some degree of remaining uncertainty of mechanism in this particular mouse model and its reproducibility and human relevance. Without an independent model validation it will be difficult to 100% confirm the mechanism and refute the chance of other cis-regulatory effects specific to their genomic manipulation that generated the Pkhd1 del 3-67 allele. This uncertainty unfortunately lowers the significance of the manuscript and makes the findings somewhat descriptive of this particular model.

Response 2.2: While we agree additional mouse lines with independently-derived deletions of various sizes that span the region might provide additional supportive evidence, we note that we had generated four independent *Pkhd1*$^{del3\text{-}67}$ founders and bred them separately to homozygosis and all had offspring with the eye phenotype (see **Response 1.1**). This strongly suggests that the effects are due to the deletion rather than other random, off-target effects. We also would like to highlight a different mouse model with a similar phenotype referenced in our report that the reviewer might have missed [12]. The authors had set out to make a transgenic mouse expressing a mutant form of xanthine dehydrogenase (XDH) but inadvertently had created a new mouse model of glaucoma when their transgene insertion caused a complicated genomic rearrangement/deletion affecting the interval between *Tfap2b* and *Pkhd1* that resulted in loss of AP-2β expression.

--the genomic position of the boundaries of the Pkhd1 deletion should be provided even if previously published

Response 2.3: We have added the requested information to the text and we have edited Figure 1 to include mouse genomic structure for this region.

--While it seems less likely, one possibility that the authors don't appear to investigate fully but could do better to exclude is that their excision of Pkhd1 exons 3-67 resulted in a larger than expected genomic disruption/rearrangement that disrupted the Tfap2b gene or its more proximal regulatory factors. Description of what sequence/probe validation confirmed the deletion of Pkhd1 exons and the presence of wild-type tfab2b on this allele

should be provided. Long-read sequencing may provide more reassurance that the allele is fully intact other then the intended deletion.

Response 2.4: We agree this is a very important point, which was also raised by Reviewer #1. Please see the detailed response to Reviewer #1 for a full explanation of how we have excluded this as a likely explanation (**Response 1.1**). Visual inspection using Integrative Genomics Viewer [4] confirms absence of reads in the intended genomic region (chr1:20,053,820-20,613,713) and no deletions directly affecting the *Tfap2b* gene.

A more deliberate demonstration of successful Tfap2b expression in other spatiotemporal conditions would alternatively be reassuring that the Tfap2b gene is not disturbed.

--single cell or tissue specific RNA seq could be informative to validate the proposed transcriptional regulation and distinguish that the lack of AP-2B staining in certain structures by immunoflourescence is indeed due to cis-regulation from the Pkhd1 genomic region as opposed to effects from different cell type/differentiation/microenvironment in the tested region as it develops abnormally.

Response 2.5: Reviewer #1 raised a similar issue (see **Response 1.3**). In brief, we showed normal expression of AP-2β in early stage *Pkhd1*$^{del3\text{-}67/del3\text{-}67}$ embryos, in later *POM* in later stage mutant embryos, and we have confirmed this is due to decreased *Tfap2b* expression in a restricted set of cells in the *Pkhd1*$^{del3\text{-}67/del3\text{-}67}$ and *Pkhd1*$^{del3\text{-}67/+}$;*Tfap2b*$^{ko/+}$ embryos by RNAscope and single nucleus RNA sequencing.

minor comments:

--Figure 3 provides publicly available data only relevant to an alternative hypothesis and perhaps could thus be supplemental.

Response 2.6: We have moved the data to the supplement, as requested (Supplementary Figure 4).

--Figure 1c would benefit from added information for the mouse allele such as chromosome number, (+/-) strand, relative genomic distances.

Response 2.7: We have modified Figure 1 to include the mouse chromosome 1 genomic interval, the strand orientation, and the genomic distances between loci.

--Expression of Pkhd1 in eye is not commonly known - often thought to be kidney, liver, pancreas specific. If more than one dataset shows transcriptomic data from eye relative to kidney that should be provided. Alternatively, if the Pkhd1 expression is not thought relevant in the proposed model which it seems no to be, that should be made more clear.

Response 2.8:

As the reviewer correctly understood, our data show significant evidence that dysregulation of *Tfap2b* expression due to genomic re-arrangement in the *Pkhd1* locus is responsible for the eye phenotype and no evidence of a direct *Pkhd1* role. In response to the reviewer's query, we compared scRNAseq *Pkhd1* expression data in eye (GSE183690, PMID:34783308), kidney (PMID: 37419448) and liver (unpublished data from our laboratory) (For Reviewers Figure 1). These data suggest that while there is minimal *Pkhd1* expression in a cluster of stromal cells that express *Tfap2b*, the level is significantly lower

than in other cell types and tissues where *Pkhd1* is known to be relevant. We have added text to the discussion to make it more clear that we do not think that *Pkhd1*-derived transcripts have a direct role in the eye phenotype.

--Supplemental Figure 6: Given the equivalency between the on-target and negative control, it is not clear that the RNA scope produces any meaningful positive or negative data on eye tissue. However, as this was to investigate an alternative hypothesis, this may be ok, although doesn't contribute to excluding the alternative hypothesis of a Pkhd1 shortened transcript playing a regulatory role.

Response 2.9: We thought it was important to formally test whether we could detect *Pkhd1* expression in the eye given that at least one database had indicated that there might be a transcript comprised of a subset of 3' exons expressed in the relevant cell types. It also was important that we include both a probe for the entire *Pkhd1* gene and one targeting 3' exons found enriched in eye cell populations with high levels of *Tfap2b* expression (Supplementary Figure 6). We elected to use RNAscope since it is a sensitive method for assessing transcript expression in a spatially-restricted manner. The fact that we saw only background with both control and specific probes means that target probe expression was below the level of detection using this method. While we can't absolutely exclude a very low expression level, we think this effectively rules out an important functional role for a *Pkhd1* transcript in the eye. As noted in response 2.8, we have made it clearer in the text of the manuscript that we do not think that *Pkhd1*-derived transcripts have a direct role in the eye phenotype.

References

1. Ishimoto, Y., et al., A novel ARPKD mouse model with near-complete deletion of the Polycystic Kidney and Hepatic Disease 1 (Pkhd1) genomic locus presents with multiple phenotypes but not renal cysts. Kidney Int, 2023.PMID:37419448

2. Vasimuddin, M., et al., Efficient Architecture-Aware Acceleration of BWA-MEM for Multicore Systems. 2019 Ieee 33rd International Parallel and Distributed Processing Symposium (Ipdps 2019), 2019: p. 314-324.PMID:WOS:000539043300030

3. Danecek, P., et al., Twelve years of SAMtools and BCFtools. Gigascience, 2021. 10(2).PMID:33590861

4. Robinson, J.T., et al., Integrative genomics viewer. Nat Biotechnol, 2011. 29(1): p. 24-6.PMID:21221095

5. Abyzov, A., et al., CNVnator: an approach to discover, genotype, and characterize typical and atypical CNVs from family and population genome sequencing. Genome Res, 2011. 21(6): p. 974-84.PMID:21324876

6. NCBI Gene ID: 21419, last updated 8-Mar-2025. (https://www.ncbi.nlm.nih.gov/gene?Db=gene&Cmd=DetailsSearch&Term=214190

7. Raap, M., et al. Transcription factor AP-2beta in development, differentiation

and tumorigenesis. Int. J. Cancer. 2021;149:1221–1227. PMID 33720400

8. EMBL-EBI Expression Atlas https://www.ebi.ac.uk/gxa/home

9. Nissirio, N., et al, Noninvasive determination of intraocular pressure (IOP) in nonsedated mice of 5 different inbred strains. *J Glaucoma* 2007. 16(1): p.57-61. PMID: 17224751

10. Chatterjee, A. et al, Central corneal thickness does not correlate with TonoLab-measured IOP in several mouse strains with single transgenic mutations of matricellular proteins *Exp Eye Res,* 2013. 115: p.115:106-112. PMID: 23806329

11. Mattapallil, M. J. ,et al, The *Rd8* Mutation of the *Crb1* Gene Is Present in Vendor Lines of C57BL/6N Mice and Embryonic Stem Cells, and Confounds Ocular Induced Mutant Phenotypes. *Invest Ophthalmol Vis Sci*, 2012, 53(6):2921–2927 (2012).

12. Barzago, M. M. et al, Generation of a new mouse model of glaucoma characterized by reduced expression of the AP-2β and AP-2δ proteins. *Sci Rep* 7, 11140 (2017). https://doi.org/10.1038/s41598-017-11752-6

13. Wu, M., et al, Transcription factor Ap2b regulates the mouse autosomal recessive polycystic kidney disease genes, Pkhd1 and Cys1. *Front Mol Biosci*, 2022, 9: 946344. https://doi.org/10.3389/fmolb.2022.946344

1. Ishimoto, Y., et al., *A novel ARPKD mouse model with near-complete deletion of the Polycystic Kidney and Hepatic Disease 1 (Pkhd1) genomic locus presents with multiple phenotypes but not renal cysts.* Kidney Int, 2023.PMID:37419448

2. Vasimuddin, M., et al., *Efficient Architecture-Aware Acceleration of BWA-MEM for Multicore Systems.* 2019 Ieee 33rd International Parallel and Distributed Processing Symposium (Ipdps 2019), 2019: p. 314-324.PMID:WOS:000539043300030
3. Danecek, P., et al., *Twelve years of SAMtools and BCFtools.* Gigascience, 2021. **10**(2).PMID:33590861
4. Robinson, J.T., et al., *Integrative genomics viewer.* Nat Biotechnol, 2011. **29**(1): p. 24-6.PMID:21221095
5. Abyzov, A., et al., *CNVnator: an approach to discover, genotype, and characterize typical and atypical CNVs from family and population genome sequencing.* Genome Res, 2011. **21**(6): p. 974-84.PMID:21324876

REVIEWERS' #2 and #4 COMMENTS ON THE REVISED MANUSCRIPT

We thank the reviewers for their comments. Below, we have pasted the comments from each reviewer in black text and added our response in red.

Reviewer #2 (Remarks to the Author):

Since the original submission, the authors have provided additional data/analyses/discussion which is largely supportive of their initial claims and satisfy my concerns.

Response 2.1b: We appreciate the reviewer's positive assessment and conclusion that our additional data satisfactorily address the original concerns.

Their rebuttal is well conceived. The implication that the region deleted in the author's Pkhd1 del 3-67 model affects expression of Tfap2b in cis, as opposed to other off-target effects from making the allele, is now well supported.

Response 2.2b: We agree with the reviewer that our data show a large deletion in the expected genomic region and no 'off-target' changes in nearby regions that could conceivably be linked to the phenotypes. We would also like to point out that the genomic deletion was achieved through Cre-mediated recombination which, unlike CRISPR models, has been widely used on the assumption of specifically targeting lox-P sequences, without requiring extensive WGS validation.

The case for a causative effect between loss of Tfap2b expression and the glaucoma phenotype reported is also strong.

Response 2.3b: We agree with the reviewer.

Together, this allows for an interesting manuscript with sufficient evidence to implicate the genomic deletion of the Pkhd1 exon 3-67 in regulation of the Tfap2b locus in a way relevant for normal eye development. Their case for this being the explanation for association of human SNPs in this region with glaucoma phenotype is strong albeit not granularly defined.

Response 2.4b: We agree with the reviewer. As discussed in more detail below, GWAS studies unequivocally associate Primary Open Angle Glaucoma (POAG) to *PKHD1*: our results support a different interpretation linking the phenotype instead to likely effects of variants near *PKHD1* on *TFAP2B* expression. A granular understanding of the mechanisms is beyond the scope of this study.

The authors propose a logical and supported model in Figure 7 and include some hypotheses as it remains undefined exactly where and how in this region the relevant interaction takes place.

The revision added analysis of WGS for the Pkhd1 del 3-67 allele to address the concern from both reviewers regarding whether anything else in LD with the Pkhd1 del 3-67 locus could provide an alternative explanation. The authors consider several possibilities that they entertained and give a thorough discussion in the rebuttal. They reviewed WGS on homozygous Pkhd1 del 3-67 mice for point mutations, CNVs, and visually inspected reads in IGV for structural variants but found no additional variants in

the region of the Tfap2b locus. The WGS data with zoomed out view from IGV now provided in Supp Figure 9, excludes any large deletion at the Tfap2b locus and I trust that inspection was done thoroughly in the Tfap2b region to consider any SNVs affecting the Tfap2b promotor or breakpoints for translocations or other disrupting variants that could be otherwise missed by WGS variant callers. It comes across looking a bit surprising that color bars in the figure could suggest heterozygous variants in this homozyogus region but I trust this is just the nature of uncurated WGS data. Figure 1 has been much improved now including genomic positions. I am satisfied with this work.
Response 2.5b: We agree with the reviewer that the raised issues have been properly addressed. As the reviewer correctly surmised, we inspected the *Tfap2b* locus thoroughly. To clear up any confusion, we have reprocessed the data from the WGS analysis to display individual sequence reads for *Tfap2b*, its promoter and known enhancer regions, and any likely enhancers that map between *Tfap2b* and *Pkhd1* (Slide set). Slide 1 has the legend identifying what is on each slide and the methods; slide 2 has a reference map of the region, and the remaining slides align the sequence reads with various key genomic elements in the region. We also clarify that this a homozygous animal and that the color code reflects read mismatches compared to the reference genome. Gray horizontal bars are reads that completely match the reference genome; some reads have mismatches that are color coded based on the called nucleotide. The bottom-line is that for every position in the reference genome, multiple reads have the correct sequence in all promoter and enhancer regions; the only possible exception is a short stretch with only 7 reads, 4 of which have multiple mismatches. But even there, every position has at least one read showing the reference nucleotide. In sum, our WGS data show that our *Pkhd1*$^{del3\text{-}67/del3\text{-}67}$ mutant mice have Genbank reference sequence across the *Tfap2b* gene, its known promoter and enhancers, and other potential enhancers identified in the interval between *Pkhd1* and *Tfap2b*.

To address another concern shared by both reviewers, the authors used RNA scope to illustrate the marked reduction in Tfap2b mRNA expression in the relevant cells of the homozygous Pkhd1 del 3-67 mice. This adds a complementary assessment of what was found by immunostaining, and is nicely convincing that Tfap2b expression is dramatically reduced at the relevant timepoint and cell type in these mice, and Figure 3 is improved to show this.

In summary, the investigation supports the claims and will be a valuable contribution to the literature.
Response 2.6b: Thank you.

minor comments:
--The first paragraph of the discussion now nicely highlights additional supporting data that an independent disruption of this locus by other investigators (who were targeting XDH) resulted in the same phenotype. This is good to highlight, but the wording may be confusing at first glance. As written, it sounded like the authors of this manuscript had intended to target XDH.
Response 2.7b: We have edited the manuscript. The changes are highlighted. “We speculate that the same mechanism likely explains the similar phenotype previously

described in mice with a complex genomic alteration induced by transgenesis[52]. Barzago et al. had set out to make a transgenic mouse expressing a mutant form of xanthine dehydrogenase (XDH) but inadvertently created a new mouse model of glaucoma when their transgene random insertion caused a complicated genomic rearrangement/deletion affecting the interval between *Tfap2b* and *Pkhd1* that resulted in loss of AP-2β expression. Our model, directly targeting the *Pkhd1* locus, also nicely explains how *Pkhd1/Tfap2b* trans-heterozygous mutants develop the phenotype".

--the edited paragraph header "Pkhd1 is not expressed in the developing eye" feels almost contradictory to the sentences that follow and provided data (supp fig 4a) which appears to show some localized expression, albeit perhaps not in the appropriate context. Perhaps the authors intended to state such a definitive conclusion in light of the clear result presented in "Figure 1 for reviewers", however that isn't part of the manuscript as is. The Figure 1 for reviewers and other scRNA seq data would benefit from clarifying the age at which they were tested if the authors consider that relevant. Nonetheless, while the investigation for a transcript consisting of exons 54-56 is not exhaustive, this seemed an unlikely possibility so it is adequate, and the authors supposition that Pkhd1 expression is not relevant seems a reasonable claim from the data.

Response 2.8b: We agree that the section heading seems contradictory. The text describes evidence for low level *Pkhd1* expression in mouse eye after birth, but not during development. We edited the heading to make this clearer. It now reads: "*Pkhd1* expression is detectable in adult, but not in the developing eye". The eye scRNAseq data presented in "Figure 1 for reviewers" is from GSE129144. While the authors of that study do not specify the age, they describe the cells are from "mature murine iris". The kidney and liver samples are from adult mice.

--Figure 7 has been improved by the added illustration (7a). However, it looks like the Tfap gene illustration was inadvertently left off (7c wild-type allele 2). This is just a typo but doesn't change interpretation.
Response 2.8b: Thank you for pointing this out. We have fixed this figure.

Reviewer #4 (Remarks to the Author):
The strongest SNP associated with POAG in GWAS studies, rs17752199, is located between Tfap2b and Pkhd1.
Response 4.1: The statement that the SNP with the highest association to Primary Open Angle Glaucoma (POAG) in the 6p12 region lies between *TFAP2B* and *PKHD1* is misleading, even if had been strictly true. rs117752199 (6:51,406,848) maps <75kb from the 3'end of *PKHD1* (6:51,480,097—6:51,952,411) but ~600kb from *TFAP2B (*6:50,786,436--6:50,815,332), and the database lists it as associated with *PKHD1* (Genome Assembly GRCh37). In fact, GWAS studies unequivocally associate Primary Open Angle Glaucoma (POAG) to *PKHD1* and not *TFAP2B*. *PKHD1* ranks 12th on the list of 141 genes associated with POAG by GWAS studies, #50 out of 117 for Open Angle Glaucoma, and #57 out of 78 for intraocular pressure (see attached

“Supplementary Table 2, Tables 1,2 for Reviewers” from the AMP-T2D database downloaded December 30/31, 2025). *TFAP2B* is not even included on any of the lists. There are also numerous SNPs with p-values less than 10^{-10} that map along the length of *PKHD1* (“Table 3 for Reviewers”). In sum, the GWAS data are far more compelling in support of an association between *PKHD1* and POAG/glaucoma than *TFAP2B* as the target gene.

It is well known that Tfap2b is involved in anterior segment development. The conditional deletion of Tfap2b in mouse cranial neural crest results in anterior segment dysgenesis and congenital glaucoma. In the current study the authors show that a large deletion that includes Pkhd1 results in anterior segment dysgenesis and congenital glaucoma with concurrent suppression of Tfap2b. They hypothesize that “that gene expression of Tfap2b is regulated by at least two spatiotemporal tissue-specific enhancers and that cis-acting genomic interactions with one of them is disrupted by the Pkhd1 del3-67 deletion.” This is consistent the genetic alterations with other ocular abnormalities. PAX6 mutations cause aniridia. The SIMO element is a distal transcriptional enhancer located within the first intron of the downstream ELP4 gene which enhances the expression of the Pax6 gene. Genetic alterations in SIMO result in loss of Pax6 expression and aniridia. In the same fashion, the Pkhd1 del3-67 deletion could be eliminating a distal regulatory region for Tfap2b.
Response 4.2: We agree that there is precedence for a model in which disruption of a regulatory element within one gene could affect expression of a distal locus. It is, however, remarkable – and novel - that the dysregulation of *Tfap2b* that we observe in our model is restricted to a very small set of cells. In fact, as we describe in the manuscript: “We examined the patterns of expression of AP-2β and Sox9 at E8 and found similar patterns of expression in pre-migratory NCCs in *Pkhd1*$^{del3\text{-}67/del3\text{-}67}$ mice and WT littermates (Figure 4a). Similarly, a day later at E9, when Sox10-positive NCCs have reached their target organs, including the eye cup, we found that AP-2β -positive NCCs had normally populated the eye in both WT and *Pkhd1*$^{del3\text{-}67/del3\text{-}67}$ mice (Figure 4b-h’). Additionally, the results showed AP-2β- positive NCCs were normally populating all other destination organs in *Pkhd1*$^{del3\text{-}67/del3\text{-}67}$ mice (Supplementary Figure 8). Collectively, these results suggest that *Pkhd1* $^{del3\text{-}67/del3\text{-}67}$ mice have normal neural crest development and AP-2β expression throughout the migrating stage, and that the NCC normally reach their target organs. However, once in the eye cup, the NCC-derived POM region no longer expresses AP-2β normally.” Our study highlights the incredibly complex and precise regulation of *Tfap2b*, a gene with broad expression that controls many diverse functions.

Unfortunately, there is no direct data identifying any regulatory elements contained inside the Pkhd1 del3-67 deletion and there is no reason to believe that there is any direct connection between Pkhd1 genetic alterations and the SNPs located in this region that modify risk to POAG.
Response 4.3: While human ARPKD is not known to be associated with POAG or anterior chamber dysgenesis, one can’t exclude a direct functional link because no prior mouse or human study has described homozygosity for a similar genetic mutation (i.e. a homozygous deletion of most of the locus). A more targeted approach to identify

regulatory elements in the *Pkhd1* locus and show conclusive evidence linking changes in these regions to altered *Tfap2b* expression in the relevant cells is itself a major, multi-year effort. One would need to generate and characterize multiple mouse lines, do exhaustive WGS to exclude potential mistargeting, and could still possibly fail to detect a true association if the wrong enhancers/sequences were changed, if there is redundancy or if the phenotypes are of low penetrance/severity.

Even if we focus just on the human data, assuming that we might be able to use cells from adult eyes, the SNPs associated with POAG in humans map along the length of the *PKHD1* interval and are associated with both increased and decreased risk. Moreover, the TAD data suggest multiple points of association between regions in *PKHD1* and *TFAP2B*. Even if we could identify a set of potential *TFAP2B* enhancer sequences across the million base pairs in the correct cell type, we would still have to identify within the haploptypes associated with the SNPs the variants actually responsible for modifying enhancer function since it is unlikely that most if any of the SNPs have direct activity. This could be many years of work for many labs. It still would take much additional effort to determine how any *PKHD1* locus variant's effects on TFAP2b expression result in POAG.

Finally, we hasten to note that there have been no published human studies that report mutations in *TFAP2B* in individuals with glaucoma or anterior chamber dysgenesis either. In fact, human mutations in *TFAP2B* are known to cause Char syndrome, whose key features are patent ductus arteriosus, hand anomalies, and facial abnormalities that include unrelated eye findings.

The effect of deleting Pkhd1 in these mice results in decreased expression of Tfap2b and this change in expression results in the observed known phenotype in the mice. However, the genetic abnormality altering Tfap2b expression in patients with POAG could easily be elsewhere, such as the region between Tfap2b and Pkhd1 or in the Tfap2b gene itself.

Response 4.4: We agree with the reviewer that not all GWAS association is going to be attributable to the mechanism we propose here. In fact, we are not aware of data showing that patients with POAG have altered *TFAP2B* expression. What we claim is: "In this study, we provide *in vivo* evidence showing that genomic elements within or near *Pkhd1* control *Tfap2b* expression in a very limited set of neural crest-derived cells in the POM, and the latter is responsible for the corneal endothelial and stromal abnormalities that were observed." Given the high association of SNPs in the region with POAG in GWAS studies, our data are consistent with this region harboring sequences that are not essential for global *Tfap2b* expression (these changes in both mice and human do not cause the same phenotypes as *Tfap2b* /*TFAP2B* deletion), but that modulate its expression in a subset of cells in which its function is required for normal anterior chamber function.

The main finding in this paper is that there may be a regulatory region for Tfap2b inside Pkhd1 but it has not been further delineated by these experiments. Where is it? What is

the mechanism? Otherwise, there is little novelty in confirming that an anterior segment dysgenesis phenotype can be caused by the loss of Tfap2b.

Response 4.5: We strongly disagree with this opinion. The evidence pointing to *Tfap2b* as a cause of ASD comes from the conditional *Tfap2b* inactivation mouse study (Ref #16, our manuscript). While this study was well-done, it relied on using a Wnt1-cre that is known to cause developmental phenotypes by ectopic Wnt activation (Lewis *et al*, Ref 1 below), which complicates interpretation of results.

Furthermore, there is strong biological evidence to that suggests *Pkhd1* itself could be important in eye development/function. As noted in our manuscript, public databases list *Pkhd1* expression in relevant cell types of the eye. In addition, *Pkhd1* encodes a ciliary protein that localizes to the primary cilium. Conditional deletion of *Ift88*, another ciliary gene associated with an ARPKD-phenotype in mice, results in a similar eye phenotype to what we describe (Ref #35 of our manuscript). The protein encoded by *Ift88* is essential for normal ciliary structure and function so its loss could either disrupt fibrocystin function or that of other ciliary proteins important for eye development. We also note that another gene associated with an ARPKD phenotype in mice is similarly linked to POAG by GWAS (#16 on the list of 141 genes; “Supplementary Table 2”).

A more indirect mechanism is also possible: SNP rs17752199 is predicted to affect a binding site for *Msx2* (“Figure 1 for Reviewers”), a transcription factor essential for normal eye development and associated with anterior angle dysgenesis (Zhao *et al*, Ref #2 below). Given the SNP’s proximity to the 3’end of *Pkhd1*, one could not exclude *PKHD1* as the target of its effects, particularly since there are no data indicating that *MSX2* regulates *TFAP2B* expression.

Another way *Pkhd1* could be responsible for an eye phenotype is as a target of *Tfap2b*. Prior studies had found that *Tfap2b* mutants can develop an ARPKD-like form of polycystic kidney disease (Ref #50, our manuscript). In fact, *TFAP2B* had been considered a candidate gene for human ARPKD prior to the identification of *PKHD1* (Wu *et al*, ref #3 below). These observations suggested a possible functional relationship between the two genes. Wu *et al* subsequently reported that *TFAP2B* may be a common regulator of *Pkhd1* and *Cys1* (another ARPKD gene) (Ref #36, our manuscript) and that *Tfap2b* deletion could result in ARPKD by disrupting *Pkhd1* and *Cys1* expression. In this framework, loss of *Tfap2b* in neural crests cells could have resulted in downregulation of *Pkhd1* in the developing eye, and **loss of *Pkhd1* could have been directly responsible for the eye abnormalities in the *Wnt1-Cre Tfap2b* model**.

In sum, the available evidence strongly suggested that *PKHD1* could have direct activity in eye development and function and in fact published data suggest that *PKHD1* could have been the effector gene downstream of *Tfap2*b that was responsible for ocular abnormalities associated with *Tfap2b, Ift88,* and possibly even *Msx2*. Our study systematically dissected the issue and provided compelling evidence that all ocular effects observed in *Pkhd1*$^{del3\text{-}67/del3\text{-}67}$ mice were likely the result of loss of *Tfap2b* expression in a small subset of cells.

With regards to the previous review of this manuscript, reviewer 1 stated “It would be important to look across this genomic interval to confirm that this was the case, and to

exclude the possibility that another variant was not inherited or introduced at the Tfap2b locus during or after Pkhd1 mutagenesis. This could be done e.g. by performing WGS and variant calling on both mutant and wild-type mice." This is a valid concern. While the authors provide some rebuttal for why they believe another variant isn't responsible, the issue cannot be eliminated without the requested WGS and variant calling on both mutant and wild-type mice, which has not been done.

Response 4.6: We acknowledge that all three reviewers had asked about any unintended or unassessed variants that could have caused the phenotype independent of the Cre-mediated deletion. As we had explained to the satisfaction of Reviewer #2, the likelihood is extremely remote since neither the Cre-line nor our original mouse lines had the phenotype, and we had four independent founder lines that had a similar eye phenotype. But we revisited our WGS analysis and found no other substantive changes, as explained above (Response 2.5b). We also disagree with the statement that "the issue cannot be eliminated without the requested WGS and variant calling on both mutant and wild-type mice, which has not been done": comparing to wild-type mice would be informative if we had multiple mismatches compared to the reference sequence and wanted to exclude that they were due to strain differences and unrelated to the phenotype. In our case, the region maps to the wild type reference genome in public databases.

We also want to remind the reviewer that the $Pkhd1^{del3\text{-}67/del3\text{-}67}$ mouse line was generated through standard Cre-lox technology. Journals require genomic proof (either PCR- or Southern blot-based methods) that the Lox P flanked interval is deleted but they do not request WGS analysis, and the community accepts that the associated phenotype is the result of the deletion. There is no reason to expect that the Cre recombinase would have introduced single/oligo base pair mutations/small deletions outside of the lox-P flanked interval. Even if it had remarkably done so by chance, we generated four $Pkhd1^{del3\text{-}67}$ founder mice and tracked the four independently-derived lines for a few generations, confirming that homozygotes in all lines had similar phenotypes. The likelihood that something other than the Cre-mediated deletion is responsible for the phenotype is infinitesimally small, and our WGS data show that none were observed affecting relevant genomic segments.

References

1. Lewis AE, et al. The widely used Wnt1-Cre transgene causes developmental phenotypes by ectopic activation of Wnt signaling. *Dev Biol*. 2013 Jul 15;379(2):229-34. doi: 10.1016/j.ydbio.2013.04.026. Epub 2013 May 3. PMID: 23648512; PMCID: PMC3804302.

2. Zhao J, *et al*. Loss of Msx2 function down-regulates the FoxE3 expression and results in anterior segment dysgenesis resembling Peters anomaly. *Am J Pathol*. 2012 Jun;180(6):2230-9. doi: 10.1016/j.ajpath.2012.02.017. Epub 2012 Apr 13. Erratum in: Am J Pathol. 2012 Jul;181(1):374. PMID: 22503753; PMCID: PMC3378853.

3. Wu, M., et al, Transcription factor Ap2b regulates the mouse autosomal recessive polycystic kidney disease genes, *Pkhd1* and *Cys1*. *Front Mol Biosci*, 2022, 9: 946344. https://doi.org/10.3389/fmolb.2022.946344

Reviewer #4 (Remarks to the Author):

The authors say in their rebuttal "In fact, GWAS studies unequivocally associate Primary Open Angle Glaucoma (POAG) to PKHD1 and not TFAP2B."

This is not correct and the authors are not citing the most recent GWAS studies. Han et al 2025 (Nature Genetics) SNPs are associated with both PKHD1 and TFAP2B. There is one significant SNP that is intronic in each gene and one that is intergenic. Their co-localization studies did not support either gene. Gene based analysis support both genes with a stronger result for TFAP2B. Fine mapping did not support either gene. Thus the GWAS data does not support one gene over the other. Additionally, only half of the time is the gene nearest to a SNP the one responsible for the effect.

We thank the reviewer for their thoughtful input.

We could not find a 2025 report by Han *et al* in *Nature Genetics* (NG) so we assume that the reviewer is referencing Han *et al's* 2023 report in NG. While we had previously referenced that study in our report, the reviewer has highlighted elements of that paper that we had not discussed as we had mostly based our analyses on information downloaded from the Common Metabolic Disease Knowledge Portal (https://md.hugeamp.org/), which aggregates, analyzes, and displays human genetic and functional genomic information from numerous studies.

We do, however, agree with the reviewer that the available multi-trait GWAS evidence does not justify assigning causality to a single gene at the *PKHD1/TFAP2B* locus, and we apologize for our over-zealous statement in our rebuttal. Our comment was in response to a previous reviewer who had implied that it was obvious that *TFAP2B* was the causal target of GWAS SNPs. We obviously did not agree: *PKHD1* was a biologically plausible candidate, SNPs near *PKHD*1 had much more significant associations with POAG (even in Figure 2 in the Han paper), and as reviewer #4 and Han *et al* note, the available data did not support one gene over the other. Our rebuttal was pushing back on the previous reviewer's declaration. Please note, however, that the manuscript was always more measured in its description of the association, and that what we had written in the manuscript was accurate.

In response to Reviewer 4's comments and suggestions, we have revised the abstract, introduction, and discussion accordingly. Specifically: we have referenced the broad *PKHD1-TFAP2B* genomic interval that is associated with POAG rather than *PKHD1*, we have tempered the conclusions in the abstract, we have added a paragraph to the discussion explicitly describing the Han *et al* results, and we note the uncertainty of which gene is implicated by the GWAS data.

What is known is that deletions of either gene in mice are associated with anterior segment developmental defects. Recessive mutations in humans in each gene have not been associated with POAG or any other form of glaucoma. They state that "What we claim is: In this study, we provide in vivo evidence showing that genomic elements within or near Pkhd1 control Tfap2b expression in a very limited set of neural crest-derived cells in the POM, and the latter is responsible for the corneal endothelial and stromal abnormalities that were observed." They should focus on presenting their results and not on explaining the human POAG GWAS association data. The proper title for the paper should be "Deletion of Pkhd1 in mice is associated with decreased Tfap2b expression and corneal abnormalities."

We agree that the primary contribution of our study is the mechanistic insight obtained from the mouse model. In response to the reviewer's suggestion, we have decreased emphasis on the human POAG GWAS data. The discussion has been revised to clarify that our study provides mechanistic insight into gene

regulation within this locus rather than direct evidence for the genetic basis of human POAG. We have also revised the title to de-emphasize the GWAS focus and to better reflect our findings.

We would like to push back gently, however, on the notion that our work is irrelevant for the human POAG GWAS association data. We agree that one must be cautious in extrapolating results from mice to humans, especially when mutations in either *PKHD1* or *TFAP2B* have not been associated with relevant eye conditions in humans. As noted in our prior comment, a previous reviewer claimed that it was self-evident that *TFAP2B* had to be the relevant target implicated by the GWAS study based on the results of neural cell conditional inactivation of *Tfap2b* in mice. As Reviewer #4 notes and the Han paper shows, the GWAS data implicate the region and do not definitively point to either gene as likely causally linked to POAG. Our data, help define a plausible regulatory mechanism and relevant cell types through which variation in the *PKHD1/TFAP2B* region could influence POAG-related phenotypes, and they illustrate why locus-level functional studies can add interpretive value beyond proximity-based annotation alone.

A useful precedent to this approach is Smemo *et al*. (*Nature* 2014, doi:10.1038/nature13138). In that paper, the authors investigated the strongest obesity GWAS signal, which mapped to noncoding variants within *FTO* introns. Prior mouse work suggested *Fto* was the effector gene; however, eQTL analyses failed to link *Fto* expression to obesity. Through a series of observations that parallel ours, the authors showed that the GWAS interval encompassed the regulatory landscape of *IRX3* at megabase distance, and that human eQTL analyses implicated *IRX3* in weight regulation. The authors then deleted *Irx3* in mice and confirmed altered weight and metabolism. They concluded their study by framing their result as a more general principle: "These observations have direct implications for how to interpret the associations characterized for thousands of noncoding variants with human disease and related quantitative traits. We observe more than 150 genomic regulatory blocks, similar to that containing *FTO* and *IRX3*, overlapping with noncoding SNPs associated with human disease and complex traits in GWAS (Methods and Supplementary Table 5), highlighting the need for a careful experimental pipeline to define the target gene(s) for each disease-associated noncoding SNP." In that same spirit, our work defines a plausible mechanistic path by which a GWAS-associated region annotated to *PKHD1* could exert disease-relevant effects through cell-type-specific regulation of *TFAP2B*.

Lastly Figure 1a is wrong. Their Manhattan plot does not reflect the association values of these loci for POAG on the website cited #37. For instance, the strongest SNPs for POAG GWAS is CDKN2B not LOXL1. Additionally, the authors should cite the primary literature here not a compilation website.

We appreciate the reviewer's concern, but there are multiple different ways to plot the information and the rank order of genes will depend on which one is selected. To assess gene–disease associations based on GWAS statistics, we used the gene-level association results ("Top gene-level associations Common variant (Ancestry: All)") for primary open-angle glaucoma available through the Common Metabolic Diseases Knowledge Portal, as shown in Figure 1a. The order will be different, however, if one chooses to order based on top single-variant association signals for POAG. In this framework, *CDKN2B* shows the strongest SNP-level association. For completeness sake, we have now added a Manhattan plot of the "Top single-variant association signals for Primary open-angle glaucoma (Ancestry: All)"—the dataset referenced by the reviewer—as Supplementary Figure 1a. We note, however, that *PKHD1* is ranked within the top signal in both datasets, appearing as the 12th strongest signal in the "Top single-variant association signals for Primary open-angle glaucoma (Ancestry: All)" and within the top 20 genes in the "Top gene-level associations Common variant (Ancestry: All)" dataset.

For Figure 1a, we have updated the analysis using the most recent data available through the Common Metabolic Diseases Knowledge Portal. We have also clarified in the figure legend that this Manhattan plot is

based on the gene-level associations for primary open-angle glaucoma derived from the “Top gene-level associations – Common variant (Ancestry: All)” dataset from the Common Metabolic Diseases Knowledge Portal.

With respect to the citation: we have revised the citation for the Common Metabolic Diseases Knowledge Portal according to the instructions of the Accelerating Medicines Partnership (https://hugeamp.org/publications.html) and have included the relevant primary literature for the GWAS studies, as requested.

REVIEWERS' COMMENTS

Reviewer #4 (Remarks to the Author): Article is acceptable in last revision.

RESPONSE:
We thank the reviewer for the positive evaluation of our manuscript and for considering the article acceptable in its current form. We appreciate the reviewer's time and valuable feedback throughout the review process.
